# Can Exergames Be Improved to Better Enhance Behavioral Adaptability in Older Adults? An Ecological Dynamics Perspective

**DOI:** 10.3389/fnagi.2021.670166

**Published:** 2021-05-28

**Authors:** Jean-Jacques Temprado

**Affiliations:** Aix-Marseille Université & CNRS, ISM UMR 7287, Institut des Sciences du Mouvement, Marseille, France

**Keywords:** exergames, cognition, brain, behavioral adaptability, ecological dynamics, complex systems

## Abstract

Finding effective training solutions to attenuate the alterations of behavior and cognition in the growing number of older adults is an important challenge for Science and Society. By offering 3D computer-simulated environments to combine perceptual-motor and cognitive exercise, exergames are promising in this respect. However, a careful analysis of meta-analytic reviews suggests that they failed to be more effective than conventional motor-cognitive training. We analyzed the reasons for this situation, and we proposed new directions to design new, conceptually grounded, exergames. Consistent with the evolutionary neuroscience approach, we contend that new solutions should better combine high level of metabolic activity with (neuro)muscular, physical, perceptual-motor, and cognitive stimulations. According to the Ecological Dynamics rationale, we assume that new exergames should act at the agent–environment scale to allow individuals to explore, discover, and adapt to immersive and informationally rich environments that should include cognitively challenging tasks, while being representative of daily living situations.

## Striving for Healthy Active Aging: A Scientific and Public Health Challenge

With the increase in life expectancy, the number of older adults suffering from declines in brain, cognitive, physical, and perceptual-motor functions also increases. Thus, an important challenge for cognitive and behavioral science is to identify the most attractive and effective solutions for attenuating the deleterious effects of aging on the brain–mind–body system (BMBS). By combining physical and cognitive exercises thanks to virtual environments and game scenarios, exergames are promising in this respect and constitute a growing market exploited by the video games and fitness industries to penetrate the field of geriatrics and rehabilitation. Introduced by the big players of the video games industry, exergames are considered as innovative solutions to improve brain functions and cognitive performance. This hypothesis remains, however, to be unequivocally demonstrated in older adults. In this *prospective paper*, on the basis of available reviews and meta-analyses, we address this issue. Our conclusion is that, until now, exergames did not keep their promises, in particular in comparison with conventional cognitive-motor training. We contend that, beyond the heterogeneity and methodological weakness of exergames studies, a more fundamental reason is that most commercial products are implicitly based on the theoretical commitments of the classic cognitive science, which considers dual-task training as the gold standard for targeting brain plasticity and cognitive functioning (Wollesen and Voelcker-Rehage, 2014; Gallou-Guyot et al., 2020; Wollesen et al., 2020). This has two important consequences. On the one hand, exergames do not fully exploit the functional relationships between perceptual-motor, physical, and cognitive domains resulting from our evolutionary history (Raichlen and Alexander, 2017). On the other hand, they do not take advantage of the resources offered by virtual reality to improve adaptive perceptual-motor behaviors, through (inter)active exploration of immersive environments. To remedy this situation, we suggest some avenues, grounded on the theoretical frameworks of evolutionary neuroscience and ecological dynamics that could inspire the design of Ecological-Exergames (E-EG).

## Exercise and Cognition: From Separate to Combined Training

For a long time, cognitive training was considered the only way to improve brain functions and cognitive performance and make older adults smarter and more able to learn faster and better (Simons et al., 2016). However, although positive training effects were observed in some studies [e.g. (Green and Bavelier, 2003, 2006; Anguera et al., 2013)], inconsistent results were also frequently reported [for meta-analyses, see (Lampit et al., 2014; Toril et al., 2014)], which has led to vivid controversies [for an overview (Simons et al., 2016)]. Progressively, they died down, thanks to the studies that have identified the effective specifications of cognitively demanding digital environments, which make video games effective to stimulate brain and cognition [for details, see (Bediou et al., 2018; Dale et al., 2020)].

In this context, the meta-analysis conducted by Colcombe and Kramer (2003) has considerably renewed the view of the relationships between exercise and cognition by showing that physical activity may also improve neuroplasticity and cognitive functioning. Then, subsequent studies contributed to elucidate the mechanisms underlying the benefits of endurance training and muscular resistance training on brain and cognitive functioning [for overviews (Voelcker-Rehage and Niemann, 2013; Herold et al., 2019; Netz, 2019)]. More recently, it has been demonstrated that complex motor skills training also improved brain plasticity and cognitive functioning [e.g. (Voelcker-Rehage et al., 2010, 2011; Niemann et al., 2014; Netz, 2019)]. For instance, available data suggested that the effects of aerobic training were magnified when aerobic effort was supported by complex motor skills [e.g. (Pesce, 2012; Diamond and Ling, 2016, 2019)]. In addition, physical-cognitive training (Fissler et al., 2013; Bamidis et al., 2015) and motor-cognitive training (Gallou-Guyot et al., 2020; Gavelin et al., 2020) were found to be more effective than separate training to improve cognitive functions [but see (Zhu et al., 2016)], for a different conclusion]. In view of these findings, exergames are expected to be more effective than conventional training (i.e., interventions supervised by a coach and not assisted by technologies) to improve behavior and cognition in older adults (Stanmore et al., 2017; Martin-Niedecken and Mekler, 2018). However, this optimistic hypothesis needs to be confirmed, in particular with respect to conventional motor-cognitive training and physical-cognitive training. This issue is addressed in the following sections.

## Are Exergames Effective for Enhancing Brain Plasticity and Cognition?

Although encouraging results were observed in several studies [e.g. (Anderson-Hanley et al., 2012; Maillot et al., 2012; Barcelos et al., 2015; Eggenberger et al., 2015; Schättin et al., 2016; Ballesteros et al., 2018)], controversial findings have also been reported as exergames were found to be effective [e.g. (Stanmore et al., 2017)], ineffective (Ordnung et al., 2017; Sala et al., 2019), or unclear (Stojan and Voelcker-Rehage, 2019), in comparison with conventional interventions. Several reasons may explain these inconsistencies. The first reason lies on the low methodological quality and the great heterogeneity of the available studies, which is reported by most reviews and meta-analyses reported (Stojan and Voelcker-Rehage, 2019; Gallou-Guyot et al., 2020; Gavelin et al., 2020). A second reason is that most studies were carried out with off-the-shelf products, which (i) lacked proper theoretical founding concepts, (ii) disregarded basic learning and training principles provided by Movement and Sport Sciences (Caserman et al., 2020) and, (iii) were neither suited for research purposes nor adapted to the specific psychological, physical, and cognitive characteristics of older adults [e.g. (Ordnung et al., 2017)]. A third reason is that the contents of digital environments and game scenarios provided by the different products were scarcely analyzed in the literature [for a noticeable exception, see (Fronza et al., 2019)], though research on action video games has demonstrated that they significantly affect the level of cognitive demands (Bediou et al., 2018; Dale et al., 2020) and that they could have more weight in the observed benefits on cognition than the physical components (Maillot et al., 2012). Thus, whether the virtual environments of the current commercial exergames (more or less) heavily load cognitive processes remains unknown. Similarly, whether the movements requested by gameplays were effectively performed by the players was never analyzed in the different studies [for a noticeable exception, see (Anders et al., 2020)]. In addition, in most studies interested in the effects of exergaming on cognition, changes in cardio-respiratory fitness and muscle strength were not analyzed. However, this should be a prerequisite for the analysis of cognitive modifications, which are presumably mediated, at least in part, by the improvement of aerobic capacities and muscle strength. Inconsistent findings have been reported in this respect in the literature. In their meta-analysis, Peng et al. (2011) concluded that exergames facilitate light-to-moderate physical activity [see (Maillot et al., 2012) for confirming evidence], but they also mentioned that participants were often below the moderate level. However, it might depend on the type of used exergame. In support of this hypothesis, using a biking exergame, Moholt et al. (2017) showed that Exergaming can be an innovative way of high-intensity training. With respect to muscle strength, although Monteiro-Junior et al. (2016) argued that, due to weight-bearing exercises, exergames might lead to effects comparable to those observed during muscular resistance training, this hypothesis was challenged by DeVries et al. (2020), who showed reported low muscular activation in balance games. A fourth reason is that, while a wide range of exergames was used to support training interventions across the different studies, their potential differences in effectiveness were scarcely assessed in the literature. This was also the case for lab-customized exergames [e.g. (Ben-Sadoun et al., 2016; Anderson-Hanley et al., 2017; Wall et al., 2018)], whose effects were never systematically compared with those of commercial products or of conventional training [for a noticeable exception, see (Willaert et al., 2020)]. Thus, the conclusions reported in most reviews on exergames were generally based on the assumption that the different exergames were all comparable in their effectiveness, which was certainly wrong.

In summary, studies on exergames predominantly aimed at describing their benefits on cognition and behavior [scarcely on brain functions (Stojan and Voelcker-Rehage, 2019)] by comparing them to a control group of more or less active individuals [e.g. (Maillot et al., 2012; Karssemeijer et al., 2019; Adcock et al., 2020)]. Few studies aimed at determining whether their benefits were larger than those of physical or cognitive training [e.g. (Martin-Niedecken and Schättin, 2020)]. On the other hand, whether exergames are equally or more effective than conventional motor-cognitive training has been scarcely addressed until now and available findings are inconsistent in this respect [e.g. see (Eggenberger et al., 2015; Htut et al., 2018) versus (Bacha et al., 2018; Schättin et al., 2018)]. A plausible reason is that the differences between the mechanisms at work in conventional motor-cognitive training and exergaming are still unclear. Monteiro-Junior et al. (2016) argued that these mechanisms could be, at least in part, similar. Specifically, neuroplasticity (i.e., neurogenesis, synaptogenesis, and angiogenesis) could be facilitated by the stimulation of neurotrophic factors (e.g. BDNF) resulting from light to moderate aerobic physical activity, while guidance effects might result from interactions with 3D virtual environments, which are hypothesized to increase the activation of the brain areas that are related to involved cognitive functions. With respect to cognitive content of commercial exergames, several authors considered that they allow delivering motor-cognitive dual-task training (MCDT) [e.g. (Wollesen and Voelcker-Rehage, 2014; Monteiro-Junior et al., 2016; Anders et al., 2018; Gallou-Guyot et al., 2020; Gavelin et al., 2020; Wollesen et al., 2020)] since additional challenges, in the form of extra tasks, such as counting, matching objects, or avoiding obstacles, are often used to add cognitive training to the physical exercise (Anders et al., 2018). These elements create dual task situations [i.e., *Thinking while moving* situations, (Herold et al., 2018)], in which the player needs to focus on two or more things simultaneously. Strikingly, few studies investigated the effects of exergames on dual-task performance [for noticeable exceptions, see (Schättin et al., 2016; Peng et al., 2020)] but, recently, Gallou-Guyot et al. (2020) compared (indirectly) conventional MCDT training and exergaming. They did not observe any superiority of the latter over the former, though some studies suggested the opposite [e.g. (Eggenberger et al., 2015)]. In addition, they reported that most of the tested exergames weakly improved physical performance, suggesting that cognitive-motor dual-task training delivered through exergames moderately loaded metabolic capacities [e.g. (Larsen et al., 2013; Anderson-Hanley et al., 2017; Levin et al., 2017; Martin-Niedecken and Mekler, 2018; Taylor et al., 2018), but see Peng et al., 2011; Maillot et al., 2012], for a different conclusion]. Consequently, one can conclude that, in most cases, exergames did not fully exploit the synergy between facilitation and guidance effects, which is considered a basic condition for observing strong benefits on brain structures and cognitive functions, when aerobic and cognitive exercise are associated (Fabel et al., 2009; Fissler et al., 2013; Raichlen and Alexander, 2017). In summary, the available literature did not demonstrate unequivocally the superiority of exergames over the other forms of training (see Table 1). These findings suggest that new exergames should be designed to improve their effectiveness relative to conventional motor-cognitive training. In the following, we propose some avenues in this respect.

**TABLE 1:** Summary table of the main findings of the literature for (i) cognitive training alone, (ii) physical/motor training alone, (iii) combined motor-cognitive and physical-cognitive training, and (iv) simultaneous, dual-task training.

Cognitive training	Conventional physical and motor training	Combined training	Dual task training
** Cognitive training is hypothesized to improve neuroplasticity and brain functioning as a result of exercises that make significant demands on specific cognitive resources. ** Appropriate, controlled, process-based intervention designs, cognitive training may lead to small or moderate gains relative to control groups, for information processing speed, visual attention, working memory, visuo-spatial abilities, and executive functions. ** With regard to transfer to other untrained tasks, cognitive multi-tasking training seems more effective, presumably since it corresponds to cognitive demands that are closer to real-life situations than those proposed in most classic cognitive training studies. ** Whether and under what conditions cognitive training and video games are effective to improve cognitive performance and favor transfer to everyday life situations remains unclear and controversial.	** Two categories of training are commonly divided into two categories: physical training (i.e., endurance and muscular resistance) and motor training, that is, the practice of complex motor skills (balance control, multi-limb coordination, mobility…). ** Endurance training mainly affects executive functions supported by the frontal and prefrontal cortex. ** Muscular resistance training improves neuro-muscular control and has a positive impact on brain plasticity, especially in the frontal lobe, which is accompanied by improvements in executive functions, in short- and long-term memories, or attention. ** All these effects are mediated by increases in neurotrophic factors, which presumably reflects the common denominator between endurance training and muscular resistance training. ** Combining aerobic and strength training leads to a greater benefit than aerobic exercise alone. ** Complex motor skill training relies on cognitive control than the highly automatic movements that are currently used in aerobic or muscular resistance training.	** Physical-cognitive and motor-cognitive training denote two forms of association between physical and cognitive exercise. ** It is well established that (i) combined training is more effective than separate training and (ii) sequential training is more effective than simultaneous training. Whether physical-motor training is more effective than motor-cognitive training is still unknown. ** Combined training also triggers general effects depending on the physical/motor components that are integrated into the training. Whether enhancement of physical and motor capacities is a pre-requisite to observe larger effects of combined training is controversial and remains to be confirmed.	** Dual-task training is hypothesized to accustom the CNS to the sharing of cognitive/attentional resources between multiple tasks. ** Most studies investigated whether healthy older adults benefit from training interventions in DT situations, with the need of balance control while standing or walking. ** The most promising approach to reach motor benefits under dual-task conditions seems to be a general dual-task intervention program, in particular under variable priority conditions. Indeed, the majority of standing and walking studies reported positive effects on standing or walking performance under dual-task conditions.

*References are mentioned in the text.*

## How Can Effectiveness of Exergames Be Improved?

According to the above considerations, new exergames should be designed to allow better combining high level of metabolic (aerobic) activity with (neuro)muscular, perceptual-motor, and cognitive stimulations, to provide the whole BMBS the multiple and synergically combined inputs that are necessary for its plastic adaptation. This hypothesis is consistent with the Adaptive Capacity Model (ACM) developed by Raichlen and Alexander (2017) in the context of the evolutionary neuroscience approach. However, a limitation of the ACM is that it endorses the mainstream approach of cognitive (neuro)science, without deeply considering the potential of virtual environments to elicit other mechanisms, at the scale of the performer–environment relationship, which underlie the way individuals perceive, explore, navigate, and, finally, act upon their surroundings in dynamic environments (Raja and Calvo, 2017). This hypothesis is consistent with the framework of ecological dynamics (ED), which may help to formalize these mechanisms and provide guidelines for designing new products.

## The Ecological Dynamics Approach

Ecological dynamics focuses on the whole BMBS, considered as a complex dynamical system (Kelso, 1995; Seifert et al., 2015) that is embedded and informationally coupled to the continuously evolving environment (Chiel and Beer, 1997). Thus, agent–environment relationship is considered the appropriate level of analysis of adaptation mechanisms. In the BMBS, the perceptual, cognitive, cardiovascular, respiratory, and motor subsystems are deeply intertwined by virtue of mutual couplings, which give rise to stable and flexible behavioral (motor) patterns that are shaped by the coalition of organismic, task, and environmental constraints (Newell, 1985; Bingham et al., 1991; Chiel and Beer, 1997). These flexible patterns make the BMBS able to adapt to the dynamic environment.

In this perspective, perception and action cannot be understood one without the other. On the perceptual side, it is assumed that information is directly available and can be picked up by individuals to attune action (Gibson, 1979). Thus, the agent would perceive affordances, i.e., action opportunities (e.g. reaching, grasping, sitting, walking, jumping, etc.) provided to the agent by the substances, surfaces, objects, and other living creatures that surround it (i.e., the environment) (Fajen et al., 2008). That means that the environment is perceived in terms of properties scaled to the performer’s motor abilities, and action, considered as an expression of cognition, is the behavioral realization of an affordance. Thus, behavioral dynamics emerge from the continuous exploration of the perceptual-motor workspace that links the performer’s perception of task constraints to the opportunities for action, given the state of the agent–environment system at each moment in time (Pacheco et al., 2019).

The idea that perception and cognition are mutually embedded and embodied in action leads to consider cognition as a virtual supervisor, distributed over the brain, mind, body, and environment, whose function is twofold. On the one hand, cognition modulates functional interactions between the different components of the BMBS, thereby allowing to shape the emerging behavioral patterns [e.g. (Zanone and Kelso, 1992; Monno et al., 2002; Taylor et al., 2018; Temprado et al., 2020)]. On the other hand, cognition supports the attunement of behavior to the perceptual-motor workspace by supervising how the individual *does something somewhere* (Araujo, 2009), that is, how it attains a functional relationship with the world by virtue of changes in information-movement coupling [e.g. (Davids et al., 2015)].

In this context, behavioral adaptability is the primary criterion for assessing individual functional states and training benefits. It can be directly or indirectly assessed through dynamical markers (Sleimen-Malkoun et al., 2014), as, for instance, directly through (i) the repertoire of behavioral patterns (Sleimen-Malkoun et al., 2013, 2014; Temprado et al., 2020) and/or of execution strategies [e.g. (Poletti et al., 2016)] that are available and effectively used by the performer; and (ii) the dynamics of switching between behavioral patterns and strategies that are observed when task constraints are increased [e.g. (Kelso, 1984; Temprado et al., 2020)]. Moreover, adaptability can also be indirectly assessed through the complex structure of the non-linear fluctuations of the output of the system under consideration [e.g. (Lipsitz, 2002; Vaillancourt and Newell, 2002)]. It has been suggested that complexity markers can be used to assess the training benefits of behavioral adaptability [e.g. (Manor and Lipsitz, 2013; Wayne et al., 2013)], but to our knowledge, this approach has not been yet applied to the study of the consequences of cognitive-motor training with exergames.

## Designing New Exergames: An Ecological Dynamics Perspective

Two main assumptions should guide the design of exergames inspired by the ecological dynamics framework (hereafter E-Exergames). First of all, the agent–environment relationship is the appropriate level at which E-Exergames must act to transform behavior and cognition. Secondly, to develop the capacity to produce adaptive behaviors, E-Exergames should allow older adults to refine or enlarge their behavioral repertoire through repeated stimulation of the synergistic relationships between cardiovascular, muscular, perceptual-motor, and cognitive subsystems, thanks to performing complex motor tasks in immersive environments. In sum, E-Exergames should be designed to allow the players learning by *exploring, discovering*, and *adapting* to dynamic environments (Rudd et al., 2020), while producing moderate to high level of cardiovascular effort and muscular force (Wall et al., 2018).

**FIGURE 1:**
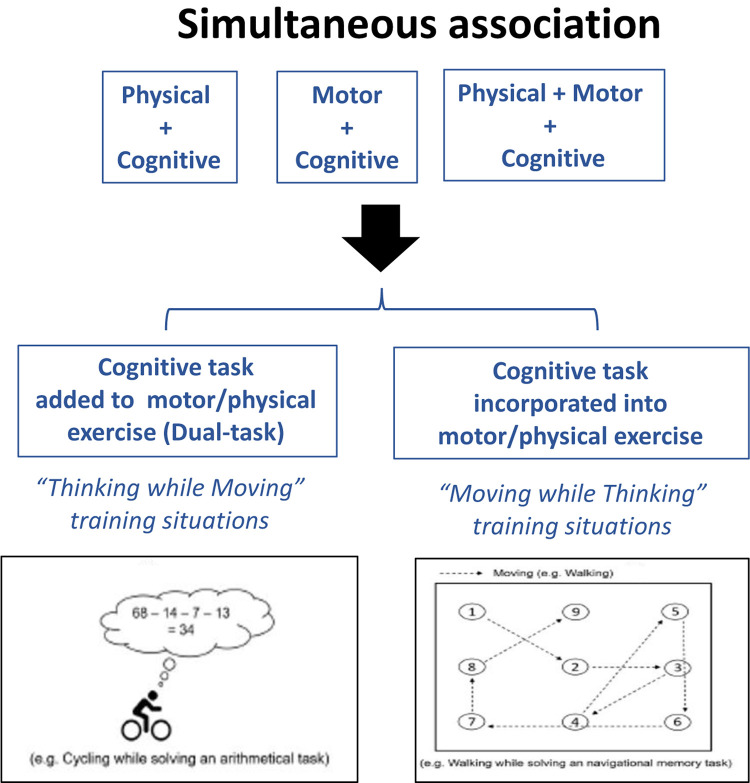
*Thinking while Moving* and *Moving while Thinking* training situations, which are hypothesized to be two different kinds training conditions. In the former, the cognitive task is simply added to the motor/physical task, while in the latter, cognition is “incorporated” into motor tasks, making them more closely related to real daily life situations [modified from Herold et al. (2018)].

To achieve these general objectives, E-Exergames should confront older adults with novel, challenging, immersive, and interactive 3D environments to facilitate the transfer of training benefits to daily living tasks. This issue is crucial and might lead to a superiority of E-Exergames over classic commercial products. Specifically, game scenarios provided by virtual environments should simulate natural activities, in emotionally salient contexts, to afford goal-oriented, exploratory motor behaviors (e.g. spatial navigation, catching, hitting, aiming, interception of mobile objects, aperture crossing, jumping, coordinating multi-limb, or controlling dynamic balance). In addition, training situations should facilitate the detection of action possibilities, to make quick and accurate decisions under time pressure, in a context of rapidly changing visual scenes [e.g. (Bediou et al., 2018; Dale et al., 2020)]. In this aim, virtual environments should enhance affordances (walkability, reachability, graspability, throwability, etc.) and allow manipulating task constraints, to afford different action possibilities and lead the exer-players to either exploit the existing patterns of their repertoire or insert new (learned) patterns (Zanone and Kelso, 1992). These guidelines are consistent with the *Moving while thinking* training situations, which have been recently hypothesized to be optimal training conditions since, in these situations, cognition is “incorporated” into motor tasks, making them more closely related to real daily life situations (Herold et al., 2018; see Figure 1).

To be more effective than commercial products, E-Exergames should combine the principles of effective cardiovascular and muscular training [e.g. (Niemann et al., 2014; Wall et al., 2018; Herold et al., 2019)], with those of perceptual and motor learning proposed by movement and training sciences [e.g. (Papegaaij and Steenbrink, 2017; Stone et al., 2019)]. In this aim, game scenarios should also be adapted to allow *task simplification*, *progressive increase in tasks difficulty*, *manipulation of information*, *modulation of constraints*, *variable conditions of practice*, and *feedback of information* about successful behaviors and goal achievements (Vaillancourt and Newell, 2002; Hristovski and Araujo, 2009; Rudd et al., 2020). Task simplification aims at making the tasks easier to achieve, while keeping present all the components of the perceptual-motor behavior (perception, decision-making, and movement patterns). Then, difficulty of the tasks must be progressively increased over the training program, in order to keep the players in the optimal flow zone. Information manipulation allows enhancing the perceptual saliency of existing affordances that invite specific functional actions needed to reach intended task goals (Raja and Calvo, 2017). It can be achieved by making some aspects of the environment more salient, while blocking others (Raja and Calvo, 2017) and/or by including additional cues, thanks to Augmented Reality (AR). In this aim, the most important information to be picked up must be identified to be subsequently used to direct the attentional focus in order to facilitate affordance perception and learning (Wulf, 2013; Becker and Fairbrother, 2019). Manipulation of constraints related to tasks, individual, or environment (Newell, 1985) allows providing variables conditions that force the exer-player to explore and discover the optimal solution (Rudd et al., 2020). Indeed, variable conditions of practice allow the exer-player to explore the perceptual-motor workspace without imposing movement solutions, until the behavioral patterns become stable and reproductible (Pacheco et al., 2019). Information feedback provided to the players will be helpful in this respect, allowing them to either reinforce their behavioral responses or explore new solutions.

Though prominently acting at the agent–environment level, E-Exergames will target cognition through the “explore–discover–adapt” process, which requires a cognitive supervision and, specifically, the involvement of executive functions, through intention and attention (Kramer and Erickson, 2007). In this context, Augmented Reality included in E-Exergames may allow educating intention (through instructions, rules, etc.) and the direction of attentional focus to the more useful variables (through the salience of some features), which, in turn, influences exploration of the environment and the detection of affordances. E-Exergames will also contribute to stimulate brain plasticity and self-repairs of the altered functional components of the BMBS, thanks to the dissipation of flows of energy and information generated by exploratory motor behavior produced at moderate-to high-intensity levels of cardiovascular effort (Kramer and Erickson, 2007; Reuter-Lorenz and Park, 2014; Raichlen and Alexander, 2017). Based on these mechanisms, extensive practice of E-Exergames could contribute to restore age-related loss of complexity of the whole brain–mind–body–behavior system that is currently associated with behavioral adaptability (Vaillancourt and Newell, 2002; Manor et al., 2010).

## Conclusion and Perspectives

During the last two decades, a number of studies aimed to assess the effects of exergaming on behavior and cognition. However, due to its great heterogeneity and methodological weaknesses, poorly informative or inconsistent findings piled up across studies, which precluded researchers to firmly conclude its superiority over conventional training.

In the present paper, we proposed new directions to design (hopefully more effective) E-Exergames, which should be based on two main pillars: on the one hand, exploiting the functional relationships between perceptual-motor, physical, and cognitive domains resulting from our evolutionary history, and on the other hand, acting at the agent–environment scale to allow individuals *to explore* the dynamic perceptual-motor workspace offered by each game situation and *to discover* and *select* appropriate actions in order *to adapt* to complex environments. In addition, to improve engagement and enjoyment, they should to create a positive flow experience for the players (Martin-Niedecken and Mekler, 2018). Then, E-Exergames would be both attractive, to encourage further meaningful engagement in active live habits, and effective to develop a wide range of complex and flexible perceptual-motor skills (Rudd et al., 2020).

To verify the hypotheses developed in the present paper, further studies are necessary, in particular to determine whether and under which conditions they are more effective, attractive, and acceptable than currently available commercial products and conventional training interventions.

## Data Availability Statement

The original contributions presented in the study are included in the article/Supplementary material, further inquiries can be directed to the corresponding author.

## Author Contributions

The author confirms being the sole contributor of this work and has approved it for publication.

## Conflict of Interest

The author declares that the research was conducted in the absence of any commercial or financial relationships that could be construed as a potential conflict of interest.
